# Poly(methyl methacrylate) bone cement composited with mineralized collagen for osteoporotic vertebral compression fractures in extremely old patients

**DOI:** 10.1093/rb/rbz045

**Published:** 2020-01-16

**Authors:** Kefeng Luo, Guoqiang Jiang, Jinjin Zhu, Bin Lu, Jiye Lu, Kai Zhang, Xiumei Wang, Fu-Zhai Cui

**Affiliations:** 1 Department of Orthopaedic Surgery, The Affiliated Hospital of Medical School of Ningbo University, Zhejiang, China; 2 Department of Orthopaedic Surgery, Sir Run Run Shaw Hospital, Zhejiang University School of Medicine, Zhejiang, China; 3 State Key Laboratory of New Ceramics and Fine Processing, School of Materials Science and Engineering, Tsinghua University, Beijing 100084, China

**Keywords:** percutaneous vertebroplasty, vertebral compression fractures, PMMA bone cement, mineralized collagen, elderly patient

## Abstract

To examine the clinical effects of a new bone cement composed of poly(methyl methacrylate) (PMMA) and mineralized collagen (MC) compared with pure PMMA bone cement in treating osteoporotic vertebral compression fractures (OVCFs) in patients aged over 80. In all, 32 cases using pure PMMA bone cement and 31 cases using MC-modified PMMA (MC-PMMA) bone cement for OVCFs between June 2014 and March 2016 were screened as PMMA group and MC-PMMA group, respectively, with an average age of over 80. The operation duration, intraoperative blood loss, hospital stay, oswestry disability index (ODI), visual analogue scale (VAS), anterior vertebral height (AVH), intermediate vertebral height (IVH) and posterior vertebral height (PVH) of injured vertebrae, vertebral computed tomography value, re-fracture rate of adjacent vertebrae, correction rate of spinal kyphotic angle and wedge-shaped vertebra angle and surgical complications were compared between the two groups. In the early post-operative period, the VAS, ODI, AVH and IVH in MC-PMMA group were comparable to those in the traditional PMMA group. Moreover, the MC-PMMA group showed better effects compared with the PMMA group 12 months after surgery. Thus, this new bone cement has superior clinic effects in the long term.

## Introduction

Generally, osteoporotic vertebral compression fractures (OVCFs) manifest as the vertebral body collapse leading to a decrease in the vertebral height [1]. These fractures produced by spinal trauma can cause severe back pain in the geriatric patient, leading to both severe movement inhibition and life quality reduction [2]. Percutaneous vertebroplasty (PVP) is widely known as the minimal invasiveness; however, its safety and efficacy for patients aged over 80 have not been established.

Filling materials for PVP, including poly(methyl methacrylate) (PMMA), are the key factors for promoting the curative efficacy; however, traditional PMMA bone cement with a relatively high elastic modulus can cause a probable increased risk of the re-collapse of treated vertebrae and the adjacent vertebrae [3]. In addition, the lack of an osteointegration ability of pure PMMA bone cement leads to loosening, dislodgement and required further surgeries [4]. Mineralized collagen (MC), with a unique nanostructure similar to natural bone, possesses good biocompatibility and osteoinductivity, which has been approved by China Food and Drug Administration and US Food and Drug Administration [5]. The new PMMA bone cement containing MC obtained the downregulated elastic modulus and significantly improved biocompatibility, which contributes to enhance the osteointegration of the injured site in both animal experiments and clinic research [6, 7].

According to our previous research, PVP with MC-modified PMMA (MC-PMMA) bone cement was effective for most patients with OVCFs [8–10]. However, one interesting phenomenon is that the effect of bone cement may be different when the patient is over 80 years old, because the bone of geriatric patients is more brittle and harder to heal than that of younger patients. Therefore, when treating elderly patients who are 80 years old or over, we still face the question of whether the PMMA modified with MC is effective for them. If we consider patients aged over 80 in the indication criteria of PVP with PMMA modified with MC, this treatment can be used in almost all patients with vertebral fractures. This determination can be made through the interpretation of clinical follow-up evaluations. In this article, we retrospectively analyzed the safety and efficacy of PVP with PMMA bone cement and MC-PMMA bone cements for patients aged 80 or over for 1-year follow-up.

## Materials and methods

### General information

The present work was approved by the ethics committee of the Affiliated Hospital of Medical School of Ningbo University. Sixty-three patients aged 80 and over with a single osteoporotic vertebral fracture, including T6 to T12 and L1 to L5, were treated by PVP and followed up from July 2016 to July 2017, as shown in Tables 1 and 2. Patients were examined by magnetic resonance imaging (Signa VH/i 1.5 T, General Electric Company, USA), preoperative vertebral X-ray (TOSHIBA, Tokyo, Japan) and computed tomography (CT) examinations (Philips Medical System, Amsterdam, Netherlands).

**Table 1:** The basic information of patient

	PMMA bone cement	MC-PMMA bone cement
Age (years)	84.54 (3.53)	83.29 (2.89)
BMD hip (T-score)	−3.08 (0.87)	−3.20 (0.98)
BMD spine (T-score)	−3.51 (0.92)	−2.59 (1.11)
Total patients/male/female	32/5/27	31/6/25
Operative time (minutes)	19.42 (2.46)	18.92 (1.78)
Volume of bone cement (ml)	4.23 (0.72)	4.79 (0.92)
Cement leakage	8	5
Operation time (minutes)	32.32 (3.21)	31.47 (2.57)
Average length of stay (days)	5.20 (1.30)	5.40 (1.40)
Follow-up duration (months)	13.72 (1.52)	14.68 (1.41)
New vertebral fracture	7	1

**Table 2:** The basic information of operative levels

Operative levels	T6	T8	T9	T10	T11	T12	L1	L2	L3	L4	L5
PMMA	2	2	2	2	5	5	2	2	5	3	2
MC-PMMA	1	4	3	3	2	2	7	4	1	3	1

Patients with OVCFs treated by PVP were firstly examined under following inclusion criteria: (i) PVP-treated single-level symptomatic OVCFs; (ii) treatment via bilateral portals; (iii) no complications after surgery; (iv) no additional postoperative trauma; (v) regular preoperative and postoperative radiologic studies; and (vi) regular antiosteoporotic treatment during the follow-up period after PVP. The exclusion criteria were as follows: (i) multiple-level PVP and (ii) non-osteoporotic vertebral compression fractures or compression fractures secondary to other factors, such as pathologic fractures due to metastasis or symptomatic hemangioma.

The patients were randomly divided into MC-PMMA group and traditional PMMA group. The MC-PMMA group included 31 patients (6 men and 25 women with an average age of 83.29). The traditional PMMA group included 32 patients (5 men and 27 women with an average age of 84.54). The general information of the two groups was comparable with no statistical significance. The bone mineral density (BMD) and other clinical data from L1 to L4 and at the hip joint were evaluated by dual-energy X-ray absorptiometry (DISCOVERY, New York, American), as shown in Table 1.

### Surgical methods

**Figure 1:**
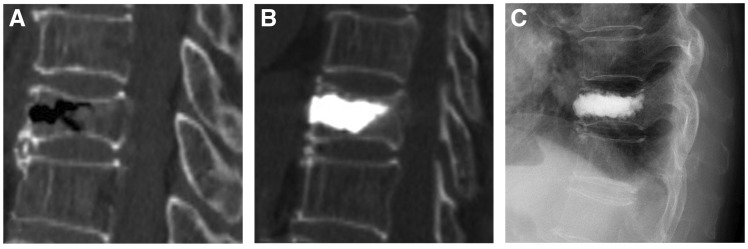
An 81-year-old male patient underwent PVP for T12 OVCFs. (**A**) Preoperative CT showed the compression fracture of the T12 vertebral body. (**B**, **C**) Lateral projection re-examination by CT and X-ray performed 3 days and 1 year postoperatively

The operation procedure was same as previously reported [8]. Briefly, the puncture needle apex reached 1/3 of the vertebral body, threading through the working channel to 1/3 of the anterior edge of the vertebral body. Positional fluoroscopy was used to confirm that the threading needle reached or slightly exceeded the middle line of the injured vertebral body. After a good puncture position was confirmed, the threading needle was removed. Then, the bone cement (PMMA bone cement or PMMA bone cement modified with MC) and the MMA monomer liquid were mixed evenly until the viscous wire-drawing stage, and the mixture was then injected. The starting time of injection was recorded, and the whole process of injecting the cement was monitored by C-arm fluoroscopy, ensuring that the bone cement infiltrated along the clearance of the trabecular bone. The injection finished after bone cement reaching the spine-like edge of the vertebra to the cortex. The time at which the injection was completed and the amount of injected bone cement were both recorded, as shown in Fig. 1.

After a 10-minute observation, the operation could then be considered finished when the activity of the patient’s lower extremities was normal and the vital signs were stable.

The PMMA bone cement (Mendec Spine; Tecres S.P.A., Verona, Italy) modified with MC consisted of 22.6 g of traditional bone cement, 3.4 g of MC (Beijing Allgens Medical Science and Technology Co., Ltd., certificated by China Food and Drug Administration and US Food and Drug Administration), and 10 ml of MMA monomer liquid. All ingredients were mixed evenly until the viscous wire-drawing stage was reached when the cement was ready for injection.

### Postoperative treatment

After the operation, the patients were required to remain in the supine position for 6 hours and then sit up after 12 hours and to walk after 24 hours. Postoperative calcium supplementation and anti-osteoporosis treatment were also given.

### Observation targets

The operative duration time and length of hospital stay of the patients were recorded. The Oswestry disability index (ODI) and visual analogue scale (VAS) were measured before the operation, 3 days after the operation and at each follow-up visit. These measurements were then used to evaluate pain relief. The VAS was used to assess the patient’s pain severity. The VAS score was measured to assess the patient’s pain severity ranging from 0 to 10. The ODI score was measured to evaluate the patient’s daily activity and degree of dysfunction. Additionally, the anterior, intermediate and posterior vertebral height were measured both before and after the operation and at each follow-up visit. Nine regions of the transverse section of pedicle of the vertebral arch were obtained according to the triangle points of the longitudinal axis and the transverse axis, and one equal circle was selected for each region. The CT value in the injured vertebra was also measured, and the average was calculated both before and after the operation. The incidence of postoperative complications was also recorded and statistically analyzed. The correction rate of spinal kyphotic angle (Cobb) and the rate of adjacent vertebral re-fracture were calculated using CT and X-rays of the affected area.

### Statistical analyses

The data were presented as means ± standard deviations (SD). Statistical analysis was evaluated by the software Statistical Product and Service Solutions (SPSS) 21.0. Independent-samples *t*-tests were applied to compare the differences between groups. Results were considered significant if *P *<* *0.05.

## Results

As shown in Table 1, the average age of the 63 patients was 81, evenly distributed in the two groups. In every group, female patients accounted for a larger proportion, of which the number was four to six times as that of male patients. The mean length of operation time of the MC-PMMA group was 19.42 ± 2.46 min and that of the traditional PMMA group was 18.92 ± 1.78 min. The mean length of hospital stay of the MC-PMMA group was 5.40 ± 1.40 days and that of PMMA group was 5.20 ± 1.30 days. All 63 patients were followed up for 12–18 months after surgery, with an average of 13.72 months in the PMMA group and 14.68 months in the MC-PMMA group. Seven patients in the PMMA group underwent adjacent vertebral re-fracture, and in the MC-PMMA group there was only one patient with vertebral re-fracture, which indicated that the MC-PMMA group had a significantly lower incidence of adjacent vertebral re-fracture than the traditional PMMA group (*P *<* *0.05). Eight patients in the PMMA group and five in the MC-PMMA group underwent bone cement leakage. The MC-PMMA group had less cement leakage cases compared with the traditional PMMA group without a significant difference (*P *>* *0.05). No bone cement was released from the posterior edge of the vertebral body into the spinal canal, and no clinical symptoms caused by cement leakage were found in either group.

**Figure 2:**
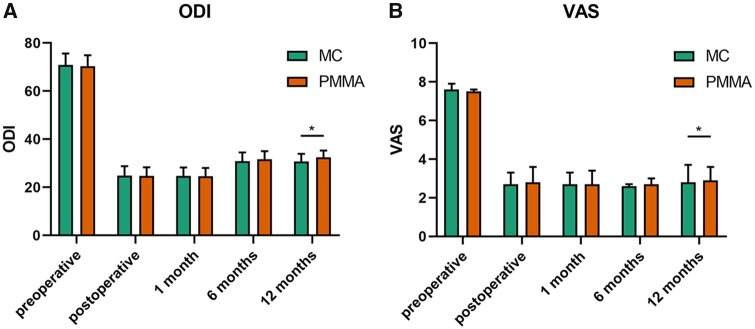
All data were expressed as the mean ROM ± SD. The ODI score (**A**) and VAS score (**B**) were evaluated and compared between MC-PMMA group and PMMA group preoperatively, postoperatively, 1 month, 6 months and 12 months postoperatively. * (*P* < 0.05) indicates a significant difference between the corresponding groups (as indicated by the horizontal bar). The VAS and ODI were evaluated by three doctors

The VAS scores and ODI scores were calculated using data gathered immediately after surgery and also at 1, 6 and 12 months after surgery. After surgery, both of the scores decreased dramatically. However, the ODI score significantly improved over time in both groups (*P *<* *0.05), especially after 6 months. The VAS score also went up slightly at 12 months. There was no significant difference between the two groups at each follow-up appointment (*P *>* *0.05) until the 12-month examination. At this time, the scores were significantly different between the two groups, as shown in Fig. 2.

**Figure 3:**
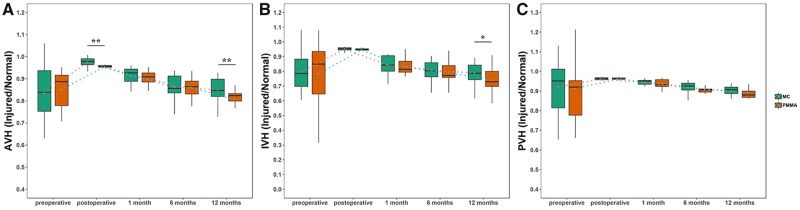
The vertebral height recovery of AVH (**A**), IVH (**B**) and PVH (**C**) preoperatively, postoperatively, 1 month, 6 months and 12 months after surgery. * Indicates a significant difference at *P *<* *0.05

The vertebral heights were evaluated according to three positions of the vertebra, which were AVH, IVH and PVH. The heights of injured vertebral bodies at each follow-up visit were significantly improved from presurgical heights in both groups (*P *<* *0.05); however, the heights of injured vertebral bodies decreased with each subsequent follow-up period in both groups, especially in the traditional group. The AVH of MC-PMMA group was significantly higher than that of PMMA group postoperatively and at 12 months (*P *<* *0.01). The IVH of MC-PMMA group was significantly higher than that of the PMMA group at 12 months (*P *<* *0.05). However, there was no significant difference between the PVH of PMMA group and MC-PMMA group at each follow-up (*P *>* *0.05) (Fig. 3).

**Figure 4:**
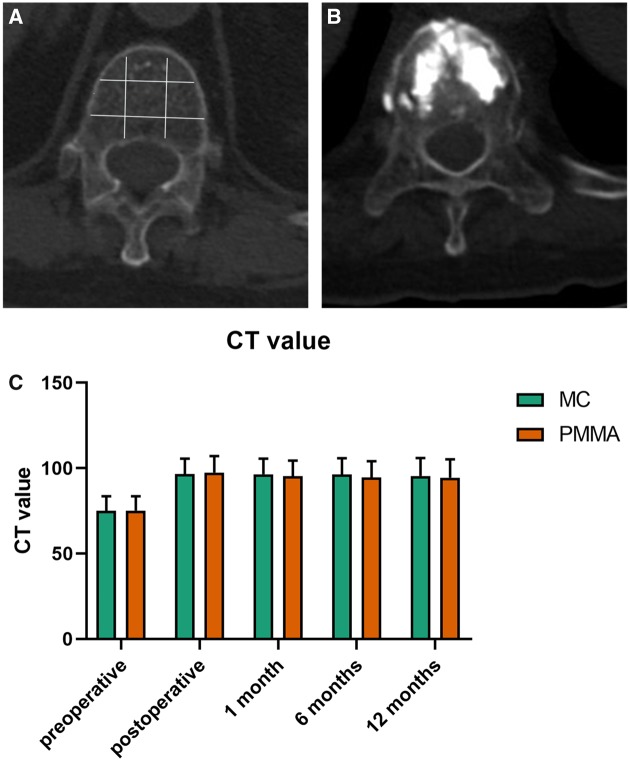
CT examination of the injured vertebra. (**A**) The measure method of CT value. (**B**) CT image of the vertebra after implantation. (**C**) The CT value of the MC-PMMA group and PMMA group 1 year after surgery

**Figure 5:**
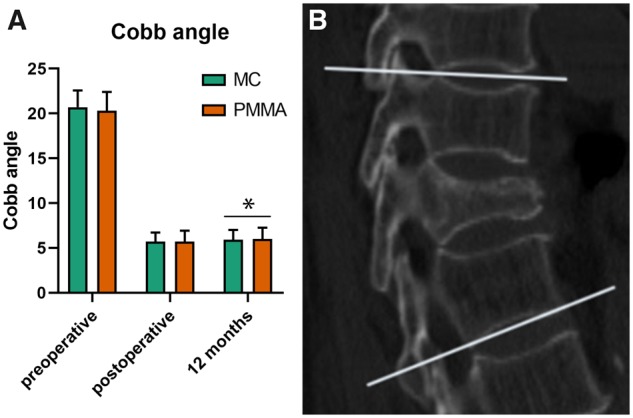
(**A**) The results of cobb angle. (**B**) The measurement method of kyphotic angle (cobb angle)

The CT value of the injured vertebra in the MC-PMMA group was significantly higher after surgery than before (*P *<* *0.05). The CT value of the injured vertebra in the traditional PMMA group was also higher after surgery than before, but the difference was not statistically significant (*P *>* *0.05), as shown in Fig. 4. The Cobb angle changed quickly after the surgery and was significantly different between the two groups at the 12-month follow-up evaluation, as shown in Fig. 5.

## Discussion

Generally, conservative treatments were applied to elderly patients with OVCFs; however, some patients could suffer from such therapy because of several risk factors that present a challenge with conservative treatment, including advanced age, dementia, pain and the presence of bone fractures. Most researchers agree that PVP is an effective way to treat OVCFs in elderly patients [11, 12]. In addition, PVP has a notable effect on the improvement of pain. Studies reported that vertebroplasty significantly reduced pain in more than 95% of patients [13]. As far as we know, no other studies demonstrated the degree of safety and efficacy of PVP using PMMA and MC-PMMA bone cement for treatment in extremely elderly patients. Therefore, our study first examined either PMMA bone cement alone or PMMA modified with MC to treat patients aged over 80 in detail PVP. In our study, we evaluated the effect of PMMA or MC-PMMA bone cement on 63 patients with OVCFs treated by PVP for 1 year according to ODI and VAS score, three vertebral height, CT value and Cobb angle. The values of ODI and VAS were significantly decreased in both groups after surgery and had an obvious increase at 12 months. After 12 months, the ODI and VAS scores, AVH, IVH and Cobb angle of MC-PMMA group showed statistically larger values than those of PMMA group, indicating that the new bone cement had a better performance in the long term when used as filling materials in PVP.

Considering the complications of PVP, bone cement leakage and adjacent vertebral fractures are the main concern. Generally, bone cement leakage arises probably because of the insufficient solidification and the fragmentation of bone cement during surgery and in use, which acts as a risk of other diseases. It has been reported that bone cement leakage occurred at a rate of up to 73% of treated vertebral bodies, in which up to 24% were venous leaks [14, 15]. Venmans *et al*. showed that the cement leaks occurred in 76% of patients undergoing PVP; however, in that article, a cement leak was described as any contrast extravasation seen on fluoroscopy during the PVP [16]. In our report, we showed that the rate of bone cement leakage of PMMA bone cement was 25%, almost the same as the above reported 24%. We also demonstrated that the rate of bone cement leakage of MC-PMMA bone cement was 16%, which was much lower than that of the PMMA bone cement leakage. The lower leakage can protect the elderly patients, who are generally weaker and susceptible to disease, from getting other related complications caused by free bone cement fragmentation, which will contribute to a more comfortable and longevity life after surgery.

Vertebral re-fractures are recurrent especially for the elderly with osteoporosis, which are usually caused by stress concentration because of mismatched elastic modulus between bone cement and surrounding bone. More recent studies have reported postoperative vertebral fractures at the rates of 15–29% [17, 18]. Soo Lee *et al.* showed that 38 among 244 treated patients (15.6%) underwent newly developed symptomatic OVCFs in the follow-up period [19]. Our results indicated that there was only one re-fracture in the MC-PMMA group, much less than the seven re-fractures in the traditional PMMA group. The reduction of treated and adjacent vertebral fractures was theoretically due to the lower modulus of elasticity of the new bone cement containing MC, which is beneficial to promoting the curative efficacy of PVP and improving patients’ life quality. The specific impact of elastic modulus on vertebral re-fractures is being studied both in theory and in animal or clinical study.

In the present study, a method of evaluating three kinds of vertebral heights, which were AVH, IVH and PVH, were adopted for analyzing the recovery of vertebra more comprehensively. Immediately after surgery, the recovery was obvious; however, all the three vertebral heights went through a downward process, which could be explained because of the compression on vertebrae when standing and walking. In addition, there were significant differences of AVH and IVH between the two groups at 12 months but no difference of PVH. Considering that posterior part of the vertebra was not susceptible to pressure, it is understandable that PMMA bone cement and MC-PMMA bone cement had similar performance. Nevertheless, anterior and intermediate part of the vertebra were easily exposed to external pressure, leading to the more serious reduction of height. At 12 months after surgery, MC-PMMA bone cement performed better in restoring the vertebral heights, which extended the using time of bone cement and improved the body gesture. The main determining factors in restoring the vertebral height in PVP surgery were the distribution and amount of bone cement [20, 21]. The distribution forms of bone cement commonly contain two types, named ‘O’ type and ‘H’ type. In the MC-PMMA group, ‘H’ type distribution was more frequent. The stress condition between bone cement and surrounding bone trabecula of ‘H’ type is more distributed than ‘O’ type because of larger contact area. Therefore, the ‘H’ type distribution of bone cement is more conductive to provide extensive support and maintain vertebral stability, which could interpret the superior effect of MC-PMMA bone cement.

In our research, we also proved that PMMA and MC-PMMA bone cement in equal volumes resulted in the same effect after surgery, but the results of the Cobb angle after 12 months showed a significant difference between the two groups. We think that MC-PMMA bone cement in the injured vertebra possessed the ability to form a stable connection with the surrounding tissue and blend with the surrounding cancellous bones more densely, providing a much firmer support [9–22].

An ideal modified PMMA bone cement for clinical practice should be operable, injectable and mechanically strong. The material should also have a reduced elastic modulus similar to natural bone and enhanced biocompatibility to promote the stress distribution and osteointegration in diseased vertebrae and cause little damage to surrounding tissues. In our study, we modified PMMA bone cement by adding MC. As discovered in previous study, MC can mix with PMMA evenly and have little effect on the injectability and operability of original PMMA bone cement which gives the substance practical clinical applications. Our recent results also showed that MC can significantly improve the biocompatibility of bone cement, promote cell adhesion and growth, guide the ingrowth of new bone tissue and enhance the osteointegration of bone cement in the injured vertebral body. Because of these qualities, the addition of MC can effectively prevent the loosening or shedding of the traditional PMMA bone cement, and also significantly enhance its safety and long-term efficacy. However, the number of cases should be larger and the follow-up time should be longer. In addition, more examinations on the patients should be performed. Moreover, multicenter studies in other hospitals and research centers should be carried out for better corroboration of our conclusions.

## Conclusions

Compared with traditional PMMA bone cement, the MC-PMMA bone cement can alleviate pain and improve vertebral height recovery. This material also has better biocompatibility, mechanical properties and biodegradability, which can be used as the first choice for filling material for OVCFs procedures in patients aged 80 and over. 
